# Anatomy-based fitting improves speech perception in noise for cochlear implant recipients with single-sided deafness

**DOI:** 10.1007/s00405-024-08984-4

**Published:** 2024-09-19

**Authors:** Anja Kurz, David Herrmann, Franz-Tassilo Müller-Graff, Johannes Voelker, Stephan Hackenberg, Kristen Rak

**Affiliations:** Department of Oto-Rhino-Laryngology, Head and Neck Surgery, Comprehensive Hearing Center, University Hospital Würzburg, Josef-Schneider-Str. 11, 97080 Würzburg, Germany

**Keywords:** Cochlear implant in single sided deafness, Anatomy-based fitting, Frequency-to-place mismatch, Speech perception

## Abstract

**Objective:**

To evaluate objective and subjective hearing outcomes in experienced cochlear implant users with single sided deafness (SSD CI) who used fitting maps created via anatomy-based fitting (ABF) and clinically-based fitting (CBF).

**Participants:**

Twelve SSD CI users with postlingual hearing loss.

**Intervention:**

OTOPLAN (Version 3. (MED-EL) was used to determine intracochlear electrode contact positions using post-operative high-resolution flat panel volume computed tomography. From these positions, the corresponding center frequencies and bandwidths were derived for each channel. These were implemented in the clinical fitting software MAESTRO to yield an ABF map individualized to each user.

**Main Outcome Measures:**

ABF and CBF maps were compared. Objective speech perception in quiet and in noise, binaural effects, and self-perceived sound quality were evaluated.

**Results:**

Significantly higher speech perception in noise scores were observed with the ABF map compared to the CBF map (mean SRT_50_: -6.49 vs. -4.8 dB SNR for the S_0_N_CI_ configuration and − 3.85 vs. -2.75 dB SNR for the S_0_N_0_ configuration). Summation and squelch effects were significantly increased with the ABF map (0.86 vs. 0.21 dB SNR for summation and 0.85 vs. -0.09 dB SNR for squelch). No improvement in speech perception in quiet or spatial release from masking were observed with the ABF map. A similar level of self-perceived sound quality was reported for each map. Upon the end of the study, all users opted to keep the ABF map. This preference was independent of the angular insertion depth of the electrode array.

**Conclusions:**

Experienced SSD CI users preferred using the ABF map, which gave them significant improvements in binaural hearing and some aspects of speech perception.

## Introduction

Single-sided deafness (SSD) is characterized by profound hearing loss in one ear and normal hearing (NH) (PTA4 ≤ 30 dB HL) in the contralateral ear [1]. Individuals with SSD can rely on the NH ear to facilitate listening in quiet. This can reduce the impact that hearing loss has on general quality of life relative to those with bilateral deafness [2]. A cochlear implant (CI) is the main treatment option to restore binaural hearing in severe-to-profound deafness. Use of a CI in SSD can improve speech perception in quiet and in noise, as well as spatial hearing, often to a greater degree than CI use in bilateral deafness [3–9]. Despite this, CI users with SSD (hereafter SSD CI) still have significantly poorer hearing performance compared to individuals with NH [5, 6, 10]. This is likely due, at least in part to the degraded spectral representation of sound delivered by a CI relative to an NH ear. Despite this, there is evidence that in SSD the signals from the CI and NH ear can be centrally integrated to produce a unified auditory representation [11].

In cases of SSD, it is plausible to posit that this integration of input from the CI and NH can be enhanced by improving the similarity of the two signals – particularly with regards to pitch. Studies which have investigated pitch perception in SSD CI users have reported large intra- and inter-participant variability in pitch matching. One plausible mechanism for this variability may be the existence of mismatches between the frequency band allocations of the array electrode contacts and the cochlear tonotopic sites at which those electrode contacts are located. Such deviations are referred to as “frequency-to-place mismatches”. These mismatches may be influenced by the angular depth of insertion of the electrode array (as measured by the depth of the most apical electrode contact) [12–18]. If frequency-to-place mismatches are not corrected for, arrays with shallower insertions can be expected to generate percepts that are shifted and compressed into the higher-frequency range. There have been several retrospective studies reporting on the correlations between frequency-to-place mismatches and speech perception outcomes [19–22].

Anatomy-based fitting (ABF) is a relatively new CI fitting paradigm that aims to reduce frequency-to-place mismatches through imaging-guided allocation of frequency bands. This is achieved via post-operative imaging combined with analysis using the OTOPLAN software of CASCINATION (Bern, Switzerland) to determine the intracochlear location and corresponding center frequency of each electrode contact. The frequency band allocations are then implemented using the MAESTRO clinical fitting software of MED-EL (Innsbruck, Austria). A previous study applied this workflow with a cohort of 10 bilateral CI users and found that the use of an ABF map was associated with improved speech perception in quiet and in noise relative to a conventional clinically based fitting (CBF) map, although the self-perceived sound quality was similar with both maps [23]. It was also shown that the willingness of participants to accept the ABF map was associated with the insertion depth of the electrode array: with deeper insertions associated with higher ABF map acceptance. ABF is useful in cases of bilateral CI because it can enhance the similarity of the signals provided by each CI, thereby potentially improving central representation and consequently yielding better hearing outcomes. For similar reasons, ABF may be useful in SSD to create a frequency allocation map that is better aligned the tonotopic organization of the contralateral (NH) ear.

To date, only a single case of ABF mapping in SSD has been presented in the literature [24]. This participant showed improved speech perception in quiet and in noise scores with an ABF map compared to a CBF map. However, it is not possible to infer from this single case the potential advantage of ABF in SSD. It is therefore necessary to evaluate the use of ABF in a larger cohort of SSD CI users. In the present study, we compared ABF and CBF maps in a cohort of 12 experienced adult SSD CI users. We evaluated speech perception in quiet and in noise, measures of binaural processing (summation, squelch and spatial release from masking), and self-perceived sound quality with the CI.

## Materials and methods

This prospective interventional study was conducted with approval from the Ethics Committee at the Medical University of Würzburg (ethics approval number: 204/20) and in accordance with the Declaration of Helsinki. All participants provided verbal and written informed consent prior to the start of the study.

### Participants

All of the following inclusion criteria applied: (1) to have a post-operative flat panel volume computed tomography (fpVCT) image with a secondary reconstruction of 99 μm [25]; (2) to be at least 18 years old at the start of the study; (3) to have postlingual-onset SSD with normal hearing on the contralateral ear. SSD is defined as a mean pure-tone average (PTA) threshold at frequencies of 0.5,1,2, and 4 kHz of ≥ 70 dB HL in the poorer ear and of ≤ 30 dB HL in the better ear (interaural threshold gap ≥ 40 dB HL) (following the SSD classification of Van de Heyning et al. [1]; 4) to have at least 6 months of experience with a MED-EL SONNET 2 or RONDO 3 audio processor; 5) to have at least ten active intracochlear electrode contacts; 6) to use the FSP, FS4, or FS4-p sound coding strategy; 7) to have either a CI-aided speech perception score of ≥ 25% on a monosyllable perception test at 65 dB SPL or a CI-aided speech reception threshold (SRT) of ≤ 20 dB SNR on a sentence-in-noise perception test; 8) to be willing and able to give feedback on the fitted map; and 9) to give their signed and dated informed consent before participating in any study-related procedures. Candidates that did not fulfill all inclusion criteria were excluded. No users of electric acoustic stimulation (EAS) CI devices were included, as the absence of residual hearing in the implanted ear was a prerequisite.

### ABF procedure

The post-operative fpVCT-SECO images were imported into the OTOPLAN software (Version 3). Cochlear duct length at level of the organ of Corti (OC) was calculated via the elliptic-circular approximation method [26]. The positions of each intracochlear electrode contact along the OC was then measured from the center of the round window. OTOPLAN Version 3 adds an adjustment of 2.5 mm to account for the presence of the cochlear hook region to ensure that these measurements do not systematically underestimate the insertion depth of the electrode contacts [27]. No further manual adjustments were then made. This information was then imported into the MAESTRO clinical fitting software (Version 9.0.5). Individual electrode contacts were displayed within the manufacturer’s pre-set frequency band distribution (70–8500 Hz) of the audio processor (SONNET2 / RONDO3). This was used as the basis of the ABF frequency band allocation scheme. Most Comfortable Levels (MCL) were adapted to reduce any unfavorable sounds. Contrary to Di Mario et al. [28], who only applied the ABF information in the software without any modification, we adapted particularly the first and second frequency band when necessary to physically match the real electrode contact to the frequency band in the audio processor.

**Fig. 1:**
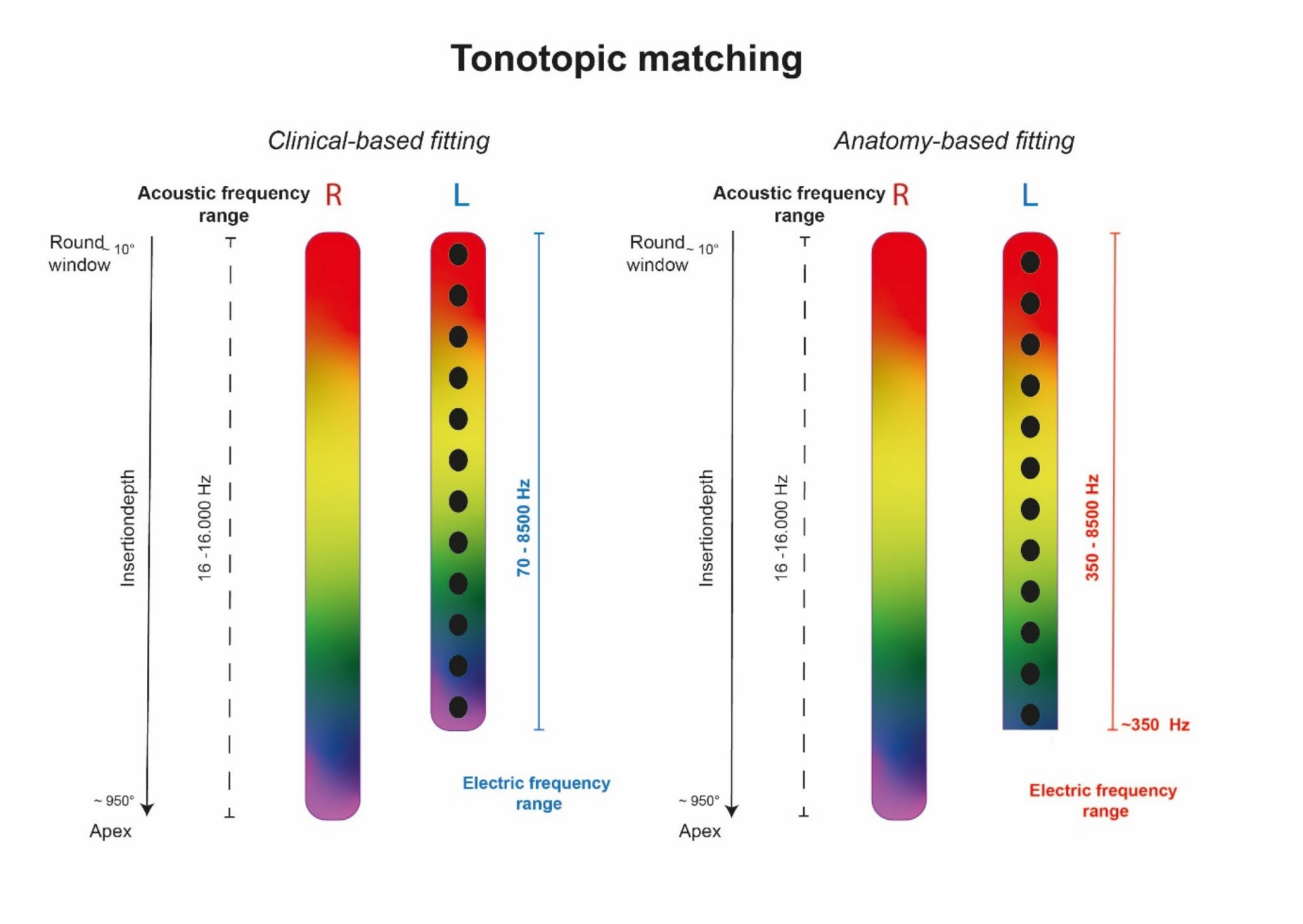
The colored bands represent the acoustic frequency distributions ranging from 16–16.000 Hz and the electric frequency distributions ranging from 70–8500 Hz in the CI audio processor for the right ear in red (R) and the left ear in blue (L). The left graph illustrates an allocation of the full electric frequency range (in light blue) from 70–8500 Hz in the CBF map, resulting in a significant mismatch at low frequencies. The right panel illustrates the ABF frequency allocation with the electric lower frequency limit (red line) in the CI map upshifted to reduce the mismatch

In some participants the tonotopic (OC) frequency of the apical electrode contact was displayed outside the first frequency band. This can happen in cases where the first contact does not reach the apex (at around 620°-720°). To cover the OC frequency of the designated electrode contact, the lower frequency limit of the filter bank in the audio processor was shifted to allow tonotopic matching as illustrated in Fig. 1. This results in a cut-off of frequencies to provide stimulation at around 350 Hz within the frequency filter, as the real electrode contact lies within this frequency region (e.g. ID 5).

### Assessment

Participants used their personal audio processers during the study. All assessments were conducted in a sound-isolated room. The participants were seated in the center of an array of nine loudspeakers (M52 Klein & Hummel, Georg Neumann GmbH, Berlin, Germany). These were equidistantly spaced in the frontal hemifield equally with a radius of 1.5 m. These were labelled 1–9 from the participant’s left to right. In this study, only loudspeakers 1 (-90° azimuth), 5 (0° azimuth), and 9 (90° azimuth) were used. The loudspeakers were connected to a pre-amplification system (Scarlett 18i20 2nd Generation, Focusrite, High Wycombe, UK). Speech perception tests were conducted using a custom program implemented in MATLAB (MathWorks, Natrick, MA, USA).

Testing took place at two intervals: a baseline interval and a one-month post-baseline interval. During baseline testing, participants used their accustomed CBF map. After baseline testing, participants were fitted using ABF and asked to use only their ABF map for 4 weeks. At the one-month post-baseline interval, testing was performed with the ABF map using the same measures as the baseline interval.

#### Speech understanding in quiet

Speech understanding in quiet was assessed using the German Freiburg Monosyllables Test material presented at 65 dB SPL [29] with the speech signal presented from the front (S_0_). Three listening conditions were assessed in free field: unilaterally to the CI via direct input; unilaterally to the NH ear with the CI off; and to both the NH ear and CI. Scores are reported as percentage correct responses. In clinical routine, masking of the contralateral ear for speech in quiet measurements is effective with earplugs and earmuffs or with an insert earphone presenting constant noise. In this study, the impact of mapping on speech perception in quiet could only be assessed by using direct audio input to preclude the effect of possible over masking. With the direct CI input, loudness matching took place prior to speech testing in quiet to ensure audibility of the streamed input. If required, the MCLs were adjusted.

#### Speech understanding in noise

Speech understanding in noise was assessed using the Oldenburg MATRIX sentence perception test with a variable noise (OLSA noise) presentation level and a fixed speech presentation level (at 65 dB SPL) [30]. The speech signals were meaningful sentences constructed from the MATRIX lists. For each stimulus-response trial, the percent of correctly identified words in each sentence was recorded. An adaptive testing procedure was used to estimate the signal-to-noise ratio (SNR) at which 50% of the words were correctly reported. The order of the presentations and the test lists were randomized to minimize the effects of training and fatigue.

To quantify the binaural hearing effects of squelch and spatial release from masking (SRM), the Oldenburg MATRIX test was conducted in two spatial configurations: S_0_N_0_ (speech and noise presented from 0° azimuth) and S_0_N_CI_ (speech presented from 0° azimuth; noise directed to the CI ear at ± 90° azimuth).

#### *Binaural* effects

Binaural effects were calculated following the protocol of van de Heyning et al. [1].

The squelch effect is the benefit of listening binaurally with a CI and NH ear relative to listening with an NH ear alone (as in untreated SSD) when the speech and noise are spatially separated:

Squelch (dB) = SRT_50_ (S_0_N_CI_) NH-only - SRT_50_ (S_0_N_CI_) CI + NH.

The summation effect is the benefit of listening binaurally with a CI and NH ear relative to listening with an NH ear alone when the speech and noise are collocated:

Summation (dB) = SRT_50_ (S_0_N_0_) NH-only - SRT_50_ (S_0_N_0_) CI + NH.

Spatial release from masking is the benefit of listening when speech and noise are spatially separated relative to when speech and noise are collocated:

SRM (dB) = SRT_50_ (S_0_N_0_) NH-only - SRT_50_ (S_0_N_CI_) CI + NH.

#### Self-perceived sound quality

Participants’ self-perceived rating of sound quality with each map was assessed using the Hearing Implant Sound Quality Index (HISQUI_19_) [31]. The HISQUI_19_ consists of 19 questions answerable on a scale from “Always”, (7 points) to “Never”, (1 point), with an additional option of “Not applicable” (0 points). The total score is obtained by adding the numerical values of all 19 questions. Results are qualified as follows: <30 points indicates “very poor sound quality”, 30–60 points indicates “poor sound quality”, 61–90 points indicates “moderate sound quality”, 91–110 points indicates “good sound quality”, and > 111 points indicates “very good sound quality”.

### Statistical analysis

Descriptive statistics (mean ± standard deviation, SD) were used to report demographic and clinical characteristics (e.g., age at testing, CI hearing experience with each ear). Descriptive statistics were also used to describe the study outcomes. Results were normally distributed according to the Kolmogorov-Smirnov test and the Shapiro-Wilk test.

Paired samples t-tests were used to assess whether the differences in test outcomes with the CBF and ABF maps were significantly different. Separate tests were conducted for each listening condition and each speech in noise configuration. *p*-values < 0.05 were regarded as significant. To account for multiple comparisons, *p*-values were adjusted using the Holm-Bonferroni method per test outcome.

Statistical analysis was implemented with SPSS Statistics (Version 25, IBM, Armonk, New York, USA). Figures were prepared with Prism (Version 8.1, GraphPad Software, San Diego, USA).

## Results

### Participants

Twelve experienced SSD CI users were included in the study. Participants had a mean age of 49.8 years (SD: 10.7 years) and had a mean CI experience of 2.9 years (SD: 1.5 years) at the time of testing. All participants had postlingual hearing loss. All participants had implants of the Mi12xx series (MED-EL). Participants’ demographics are presented in Table 1.

**Table 1:** Participants’ demographic and clinical characteristics. Impl. Side, implantation side; CI exp., CI experience; AID, angular insertion depth, measured as the insertion depth of the apical electrode contact E1; a Value, B value, H value and estimated Cochlear Duct length (CDL) in mm

Subject	Age (years)	Impl. side	CI exp. (years)	Electrode array	AID	A Value (mm)	B Value (mm)	H Value (mm)	Est. CDL (mm)
ID01	32	Right	3.0	FLEXSOFT	650.0°	10.3	7.3	3.9	39.5
ID02	40	Left	3.0	FLEXSOFT	670.8°	9.2	7.2	3.9	37.3
ID03	59	Left	1.5	Standard	614.0°	9.2	7.2	3.4	37.2
ID04	40	Left	4.5	Standard	784.8°	9.2	7.6	4.3	38.8
ID05	58	Left	6.0	FLEX24	500.7°	10.2	6.8	4.0	37.2
ID06	57	Right	2.0	FLEXSOFT	558.2°	10.1	7.6	3.8	40.2
ID07	37	Right	1.5	FLEX28	504.6°	10.2	7.5	3.4	39.9
ID08	41	Left	3.0	FLEX28	614.6°	8.9	6.4	3.6	33.8
ID09	56	Right	1.0	Standard	709.3°	8.9	6.8	3.9	35.4
ID10	63	Left	3.0	FLEX28	626.5°	8.8	6.8	3.7	35.4
ID11	51	Right	5.0	FLEX28	580.7°	9.9	7.6	3.8	39.7
ID12	64	Right	1.5	FLEXSOFT	724.7°	9.2	7.1	3.9	36.8

For each participant, the relationships between the tonotopic frequencies of each electrode contact measured with OTOPLAN (est. Freq. OC) and the center frequencies assigned to each electrode contact according to the CBF and ABF maps are given in table 2.

**Table 2:** Estimated angular insertion depths (AID) and tonotopic frequencies (est. Freq. OC, hz) of each electrode contact based on the OC measurements are given above for each participant. Center frequencies of the clinical based fitting (CBF) maps and anatomy based fitting (ABF) maps are given below

		Overview of Otoplan vs. MAESTRO Frequency allocation											
Participant		C1	C2	C3	C4	C5	C6	C7	C8	C9	C10	C11	C12
ID01	AID (°)	650.0°	581.9°	492.8°	393.2°	323.2°	376.7°	229.5°	180.8°	134.1°	90.8°	51.2°	17.5°
	Est. Freq. OC (Hz)	160.5	265.55	457	789.2	1190.8	1611.6	2220	3093.9	4353.2	6180.4	8846.7	12480.4
	Center Freq. CBF (Hz) (Hz)/Hz)	Deact.	186	308	474	698	1002	1418	1986	2766	3836	5307	7329
	Center Freq. ABF (Hz)	Deact.	208	396	680	1098	1638	2258	2966	3801	4818	6090	7649
ID02	AID (°)	670.8°	604.5°	542.2°	472.8°	387.2°	318.9°	266.0°	220.5°	174.9°	129.3°	87.8°	51.0°
	Est. Freq. OC (Hz)	135.1	226.4	341.4	507.9	810.4	1212.5	1715.4	2333.9	3186.2	4455.6	6248.1	8717.7
	Center Freq. CB (Hz)	120	235	384	580	836	1175	1624	2222	3020	4084	5507	7410
	Center Freq. ABF (Hz)	128	256	412	608	854	1216	1722	2364	3175	4218	5606	7448
ID03	AID (°)	614.0°	534.9°	468.4°	400.2°	320.4°	257.3°	208.7°	168.6°	131.2°	96.2°	61.5°	27.4°
	Est. Freq. OC (Hz)	211.4	355.9	520	753.0	1201.1	1818.7	2526.0	3328.8	4388.1	5809.9	7889.2	11022.7
	Center Freq. CBF (Hz)	126	254	425	655	966	1386	1960	2746	3822	5298	7326	Deact.
	Center Freq. ABF (Hz)	142	306	517	794	1214	1810	2522	3347	4380	5730	7498	Deact.
ID04	AID (°)	784.8°	676.1°	580.2°	508.1°	447.4°	381.5°	307.1°	238.7°	192.4°	152.7°	117.4°	82.8°
	Est. Freq. OC (Hz)	39.4	129.2	268.6	418.0	585.5	840.9	1315.3	2078.8	2845.1	3770.7	4939.8	6594.4
	Center Freq. CBF (Hz)	120	235	384	580	836	1175	1624	2222	3020	4084	5507	7410
	Center Freq. ABF (Hz)	98	178	303	457	655	946	1391	2038	2872	3944	5410	7382
ID05	AID (°)	500.7°	443.1°	386.6°	329.8°	267.0°	207.8°	165.2°	140.4°	109.5°	81.3°	49.3°	21.6°
	Est. Freq. OC (Hz)	435.0	596.2	812.5	1133.2	1703.3	2540.5	3411.8	4091.5	5207.6	6607	8857	11725.7
	Center Freq. CBF (Hz)	126	254	425	655	966	1386	1960	2746	3822	5298	7326	Deact.
	Center Freq. ABF (Hz)	404	552	803	1174	1734	2512	3292	4070	5030	6219	7688	Deact.
ID06	AID (°)	558.2°	488.5°	422.4°	352.9°	297.7°	248.1°	198.6°	155.6°	116.0°	79.3°	42.1°	10.5°
	Est. Freq. OC(Hz)	310.7	468.9	673.3	996.6	1405.5	1961.9	2745.3	3718.2	5033.7	6859.7	9707.3	13,552
	Center Freq. CBF (Hz)	120	235	384	580	836	1175	1624	2222	3020	4084	5507	7410
	Center Freq. ABF (Hz)	190	352	570	867	1222	1642	2243	2956	3744	4742	6005	7606
ID07	AID (°)	504.6°	442.5°	384.3°	324.7°	269.1°	221.9°	179.2°	144.6°	111.8°	80.3°	49.1°	22.6°
	Est. Freq. OC (Hz)	428.3	603.2	829.9	1180.7	1699.9	2341.0	3134.8	4028.4	5195.7	6781.8	9049.4	11844.9
	Center Freq. CBF (Hz)	126	254	425	655	966	1386	1960	2746	3822	5298	7326	Deact.
	Center Freq. ABF (Hz)	316	481	733	1052	1435	1918	2584	3430	4456	5789	7521	Deact.
ID08	AID (°)	614.6°	554.8°	498.5°	419.4°	320.0°	258.7°	209.5°	168.6°	130.7°	94.6°	57.5°	23.1°
	Est. Freq. OC (Hz)	208.9	313.4	435.5	669.4	1185.4	1769.9	2461.1	3256.4	4301.6	5736.8	7959.9	11180.6
	Center Freq. CBF (Hz)	120	235	384	580	836	1175	1624	2222	3020	4084	5507	7410
	Center Freq. ABF (Hz)	140	253	436	788	1212	1656	2200	2892	3740	4764	6039	7626
ID09	AID (°)	709.3°	616.3°	524.7°	446.7°	368.6°	293.0°	234.1°	184.0°	135.8°	87.6°	43.1°	14.0°
	Est. Freq. OC (Hz)	95.8	207.1	376.7	581.8	893.0	1419.5	2107.0	2955.1	4184.5	6166.0	9267.4	12517.9
	Center Freq. CBF (Hz)	120	235	384	580	836	1175	1624	2222	3020	4084	5507	7410
	Center Freq. ABF (Hz)	106	212	376	600	933	1436	2128	2982	3928	4956	6179	7679
ID10	AID (°)	626.5°	563.7°	503.3°	428.8°	353.7°	295.3°	246.5°	200.5°	156.7°	117.8°	79.5°	40.5°
	Est. Freq. OC (Hz)	192.2	297.0	425.8	640.6	974.1	1398.8	1937.8	2642.3	3584.4	4809.1	6618.0	9508.4
	Center Freq. CBF (Hz)	120	235	384	580	836	1175	1624	2222	3020	4084	5507	7410
	Center Freq. ABF (Hz)	134	276	450	668	978	1406	1954	2670	3522	4541	5854	7546
ID11	AID (°)	570.7°	503.2°	445.9°	389.9°	334.4°	280.8°	226.1°	181.8°	144.8°	112.7°	83.5°	54.6°
	Est. Freq. OC (Hz)	286.2	430.5	591.9	804.0	1112.4	1569.7	2273.6	3076.7	4018.4	5156.0	6587.7	8577.9
	Center Freq. CBF (Hz)	120	235	384	580	836	1175	1624	2222	3020	4084	5507	7410
	Center Freq. ABF (Hz)	138	288	478	718	1002	1362	1856	2542	3392	4420	5762	7510
ID12	AID (°)	724.7°	652.8°	572.0°	474.8°	393.4°	334.8°	284.8°	226.0°	170.4°	124.3°	84.9°	46.5°
	Est. Freq. OC (Hz)	82.4	156.7	282.8	502.2	780.9	1097.5	1509.7	2242.2	3279.0	4616.3	6385.2	9071.6
	Center Freq. CBF (Hz)	Deact.	157	282	450	678	985	1403	1974	2756	3830	5302	7328
	Center Freq. ABF (Hz)	Deact.	158	302	506	774	1106	1564	2276	3160	4204	5594	7444

**Fig. 2:**
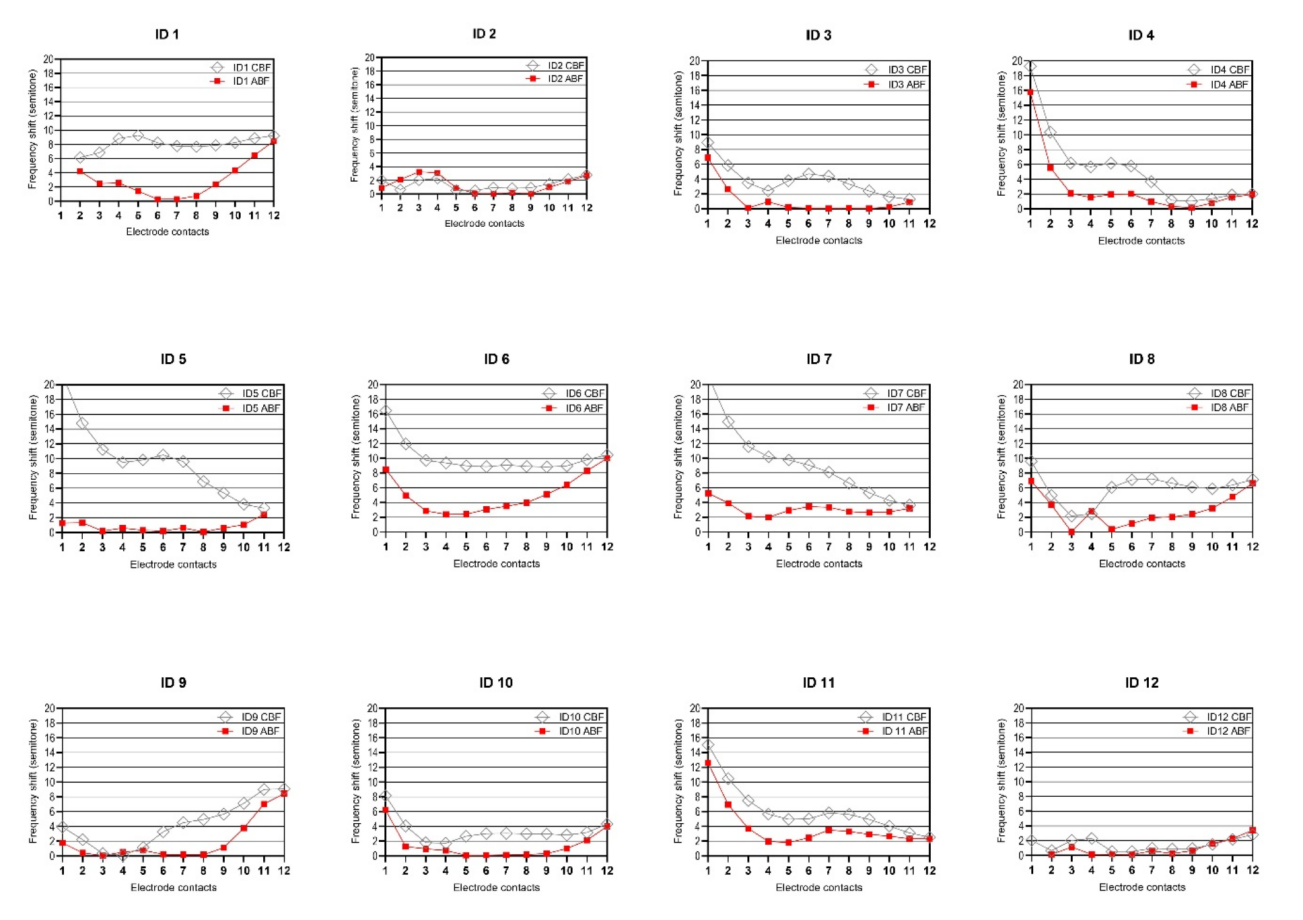
Mismatch in semitones from the post-operatively determined tonotopic frequency of each electrode contact with the CBF map (grey) and the ABF map (red) for each participant

Individual frequency deviations in center frequency with the CBF and ABF maps relative to the estimated tonotopic frequency locations of each contact are shown in Fig. 2.

A correlation between the CBF/ABF mapping and the insertion depth revealed that a significant different exists with the CBF mapping in the apical and medial electrode contacts, but not in the basal contacts (apical: electrode contacts 1–4; medial: electrode contacts 5–8, and basal: electrode contacts 9–12). With ABF, the deviation to the tonotopic frequency was not significant, implying that higher precision in tonotopic stimulation can be provided with ABF mapping.

CBF map (apical): *r*= -0.643; R^2^ = 0.414; *p* = 0.024 (*). ABF map (apical): *r* = 0.004; R² = 1.737e-005; *p* = 0.989; (Cohen´s q = 0.768). CBF map (medial): *r*= -0.694; R² = 0.482; *p* = 0.012 (*). ABF map (medial): *r*= -0.447; R²= 0.2004; *p* = 0.2004; (Cohen´s q = 0.376). ABF map (basal): *r* =-0.223; R² = 0.050; *p* = 0.484; Cohen´s q = 0.086).

### Speech understanding in quiet

Mean (± SD) scores for the CI-only listening condition were 72.1% (± 13.6%) with the CBF map and 72.7% (± 13.9%) with the ABF map. The mean (± SD) scores for the NH-only listening condition at baseline were 95.0% (± 4.3%) with the CBF map and at 1 month afterwards 96.0% (± 4.9%) with the ABF map. Test learning effects with the NH ear did not affect further benefits seen with the ABF mapping. The mean (± SD) scores for the CI + NH listening condition were 95.8% (± 3.9%) with the CBF map and 97.7% (± 2.0%) the ABF map (see Table 3).

**Table 3:** Speech perception in quiet scores. Participants were evaluated separately with the CBF and ABF maps under the three different listening conditions. Scores are presented as percent correct

	CI-only		NH-only		CI + NH	
**Subject ID**	**CBF**	**ABF**	**CBF**	**ABF**	**CBF**	**ABF**
ID01	77.5	62.5	92.5	85.0	100.0	97.5
ID02	82.5	85.0	92.5	97.5	92.5	95.0
ID03	80.0	75.0	87.5	90.0	87.5	97.5
ID04	82.5	77.5	100.0	97.5	97.5	95.0
ID05	47.5	45.0	100.0	90.0	97.5	97.5
ID06	92.5	82.5	95.0	100.0	92.5	100.0
ID07	75.0	85.0	95.0	100.0	100.0	100.0
ID08	62.5	72.5	100.0	97.5	100.0	100.0
ID09	57.5	57.5	95.0	97.5	92.5	100.0
ID10	55.0	57.5	92.5	97.5	97.5	97.5
ID11	70.0	85.0	100.0	100.0	95.0	95.0
ID12	82.5	87.5	90.0	100.0	97.5	97.5

No significant differences were observed between the mean speech perception in quiet scores with the CBF map and the ABF map in any listening condition: CI-only (t=-0.248; df = 11; *p* = 0.81), NH-only (t=-0.624; df = 11; *p* = 0.55), and CI + NH (t=-1.567; df = 11; *p* = 0.15).

### Speech perception in noise

#### S0NCI configuration

**Fig. 3:**
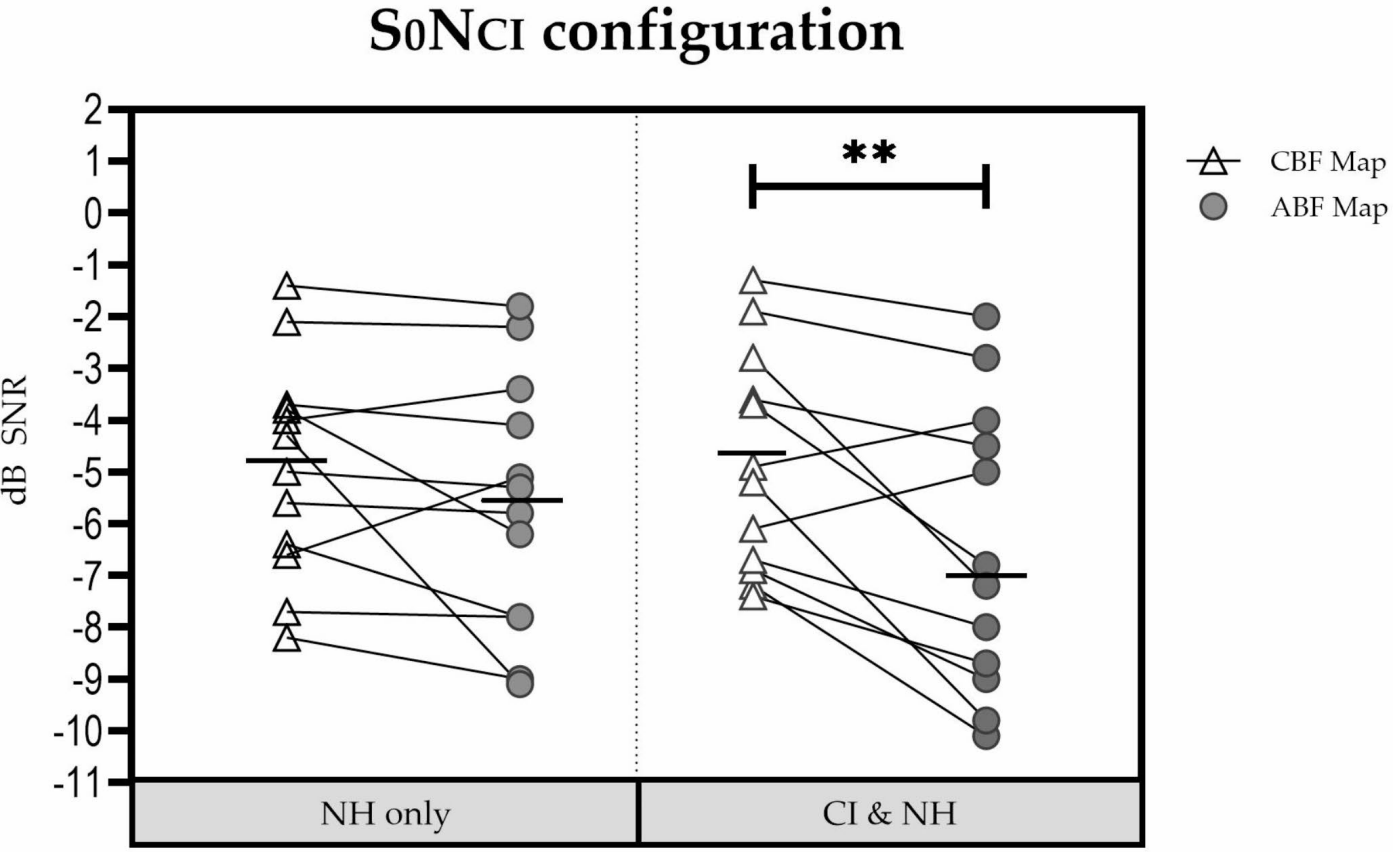
SRT_50_ values for the S_0_N_CI_ configuration for each participant with each listening condition. The horizontal bold line represents the mean. ** Significant difference (*p* < 0.01)

Individual results in the S_0_N_CI_ configuration are shown in Fig. 3.

In the NH-only listening condition, the mean (± SD) SRT_50_ was − 4.9 (± 2.09) dB SNR at baseline and − 5.6 (± 2.47) dB SNR when tested 1 months afterwards. This difference was not significant (t = 1.591; df = 11; *p* = 0.14).

In the CI + NH listening condition, the mean (± SD) SRT_50_ was − 4.8 dB (± 2.13) dB SNR with the CBF map and − 6.49 dB (± 2.76) dB SNR with the ABF map. This difference was significant in favor of the ABF map (t = 3.203; df = 11; *p* = 0.008).

#### S0N0 configuration

**Fig. 4:**
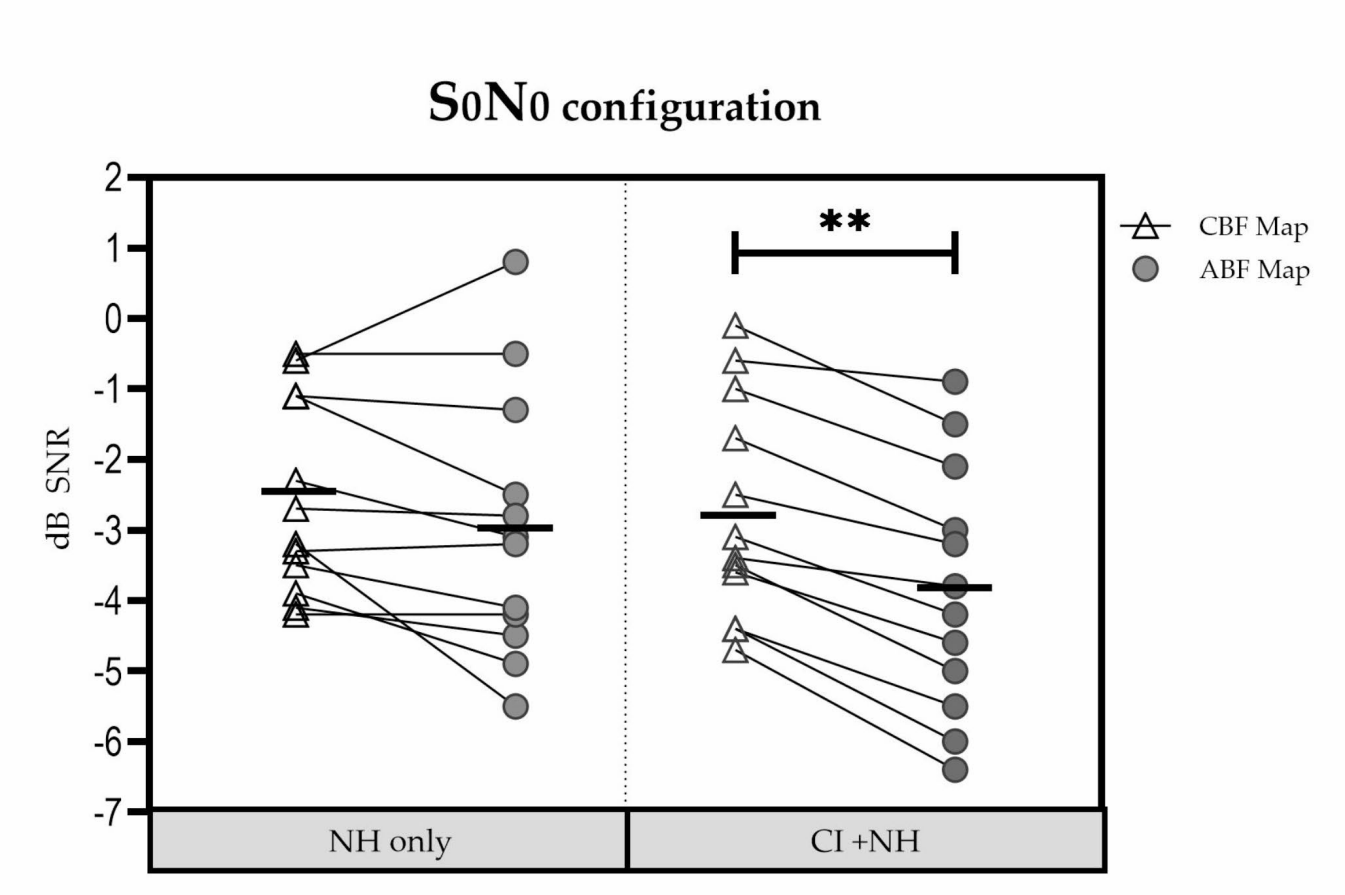
SRT_50_ values for the S_0_N_0_ configuration for each participant with each listening condition. The horizontal bold line depicts the mean. ** Significant difference (*p* < 0.01)

Individual results in the S_0_N_0_ configuration are shown in Fig. 4.

In the NH-only listening condition, the mean (± SD) SRT_50_ was − 2.54 (± 1.38) dB SNR at baseline and − 2.98 (± 1.87) dB SNR 1 months afterwards. This difference was not significant (t = 1.683; df = 11; *p* = 0.12).

In the CI + NH listening condition, the mean (± SD) SRT_50_ was − 2.75 (± 1.56) dB SNR with the CBF map and − 3.85 (± 1.76) dB SNR with the ABF map. This difference was significant in favor of the ABF map (t = 8.521; df = 11; *p* < 0.0001).

#### Binaural effects

**Fig. 5:**
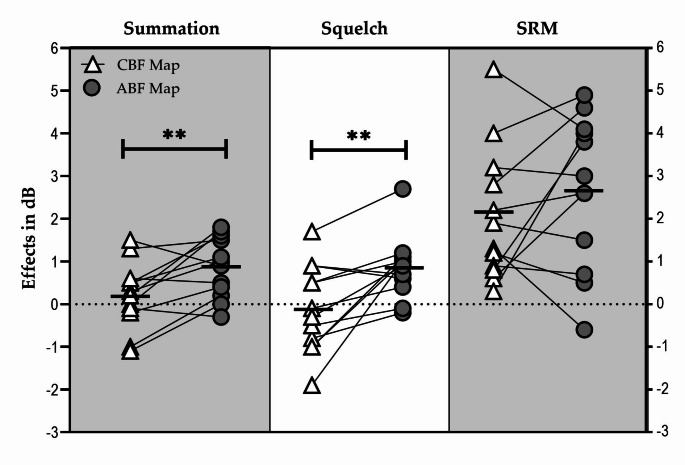
Participant-level calculated binaural effects with each map. The horizontal bold line depicts the mean. ** *S*ignificant difference (*p* < 0.01)

The calculated binaural effects per participant with each map are shown in Fig. 5.

The mean (± SD) summation effect was 0.21 (± 0.78) dB SNR with the CBF map and 0.86 (± 0.71) dB SNR with the ABF map. This difference was significant in favor of the ABF map (t=-3.149; df = 11; *p* = 0.009).

The mean (± SD) squelch effect was − 0.09 (± 1.02) dB SNR with the CBF map and 0.85 (± 0.74) dB SNR with the ABF map. This difference was significant in favor of the ABF map (t = 3.440; df = 11; *p* = 0.005).

The mean (± SD) SRM was 2.1 (± 1.56) dB SNR with the CBF map and 2.64 (± 1.77) dB SNR with the ABF map. This difference was not significant (t=-1.165, df = 11; *p* = 0.27).

### Self-perceived sound quality

**Fig. 6:**
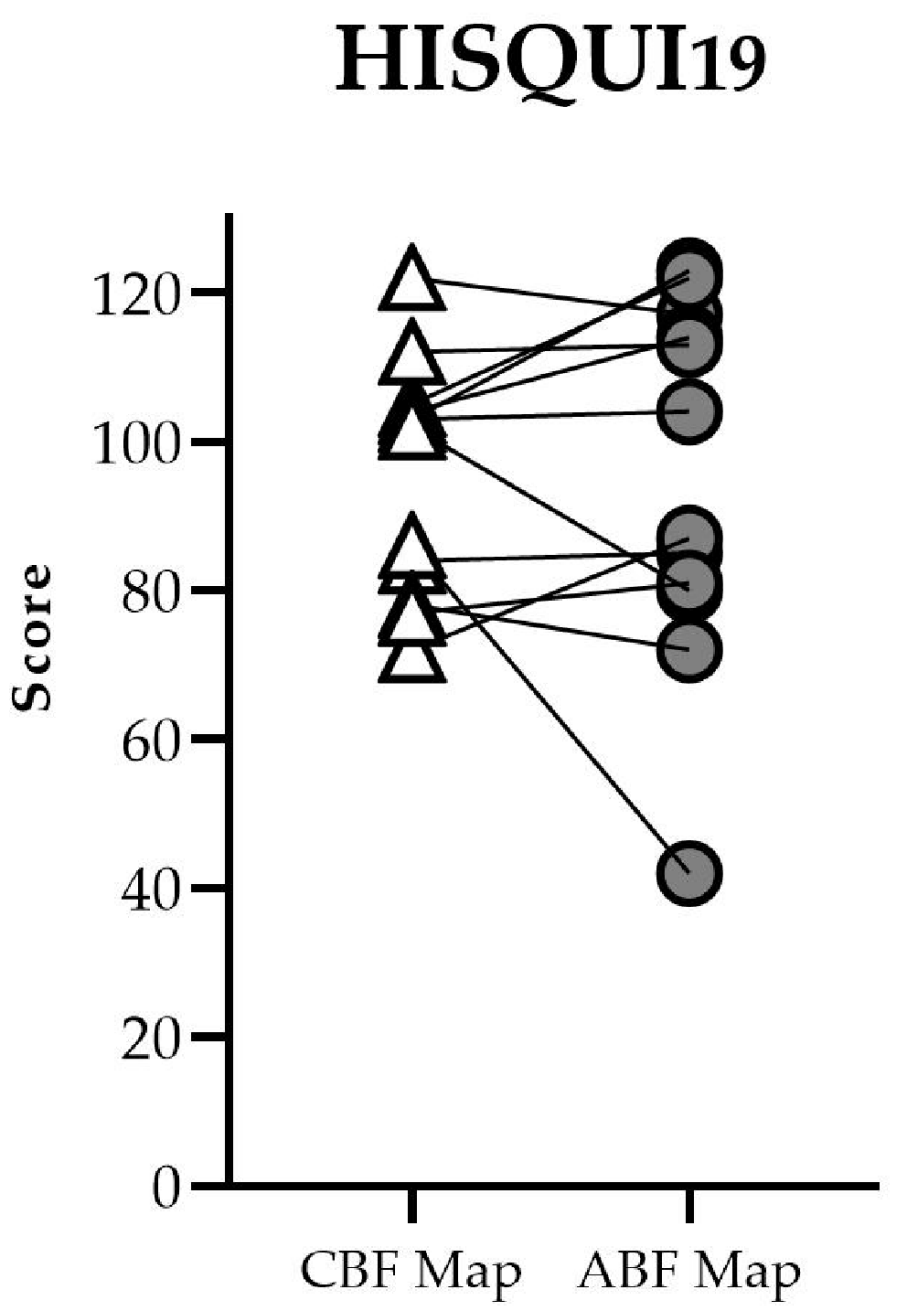
Individual HISQUI_19_ total scores for the CBF and ABF maps in the CI + NH listening condition. Higher total scores indicate higher self-perceived sound quality

Total scores on the HISQUI_19_ with each map are given in Fig. 6.

The mean (± SD) total score was 95.7 (± 15.7) points for the CBF map and 95.0 (± 24.6) for the ABF map. This difference was not significant (t=-1.165, df = 11; *p* = 0.27).

Total scores were high overall, with all but one rating categorized as “moderate”, “good”, or “very good sound quality”. 8 of 12 participants had a higher total score with the ABF map than with the CBF map in the CI + NH condition. Nevertheless, participants tended to give similar ratings for both maps.

Two participants reported a decline in sound quality with the ABF map (“moderate” to “poor” for participant 3 and “good” to “moderate” for participant 9). Two other participants reported a decline in sound quality, but in neither case was this decline large enough to change the categorization of the total score.

## Discussion

The aim of this study was to test if CI frequency allocation maps generated through ABF lead to improved speech understanding and self-perceived sound quality in adults who are experienced CI users. This hypothesis was partly validated: ABF maps significantly improved speech perception in noise in two speech-noise spatial configurations. Speech perception in quiet was not improved with the ABF map in either the CI-only or CI + NH listening condition. For the CI + NH condition, speech perception in quiet scores had a strong ceiling effect with both maps (95.8% for CBF and 97.7% for ABF). The ABF map significantly improved summation and squelch effects but did not significantly improve spatial release from masking. Most participants’ self-perceived sound quality was similarly high with both the ABF and CBF maps. The majority of the cohort (8/12) expressed a slight preference for the sound quality of the ABF map, but the differences were small in magnitude.

As the frequency mismatch calculated in semitones (Fig. 2) displays, the mapping with both CBF vs. ABF in ID 2, ID 10 and ID12 reaches a maximum deviation of 4 semitones. As the AID of E1 for this subjects lies at 650° (ID2); 626.5° (ID10) and 724° (ID12) we assume that deep insertions are beneficial to reduce the frequency- to- place mismatch particularly in SSD subjects, regardless of the applied mapping approach. This is further supported by the statistical analysis showing that insertion depth has a relevant influence on the mapping, especially in the apical and medial area, but not in the basal area. Nevertheless, our data show that the achievable precision with ABF mapping is higher in order to reduce the frequency-to place mismatch especially in the apical and medial frequencies.

To improve sound quality and acceptance with ABF mapping, the first electrode contact in participant ID01 an ID12 had to be deactivated. Figure 2 further shows that the frequency shift is huge and highly variable for all other participants irrespective of their electrode length and AID of E1. Overall, the frequency shift to the estimated electrode contacts are substantially reduced with ABF mapping.

Our group has previously tested ABF maps in bilateral CI users [23]. In that study, improved binaural processing was observed and associated with improved speech understanding in quiet and in noise in participants with unequal electrode array insertion depths and different electrode array lengths. We found that participants’ likelihood of preferring the ABF map was related to the angular insertion depth of their electrode array. More specifically, it was found that when the most apical electrode contact (E1) lay at a cochlear site corresponding to a tonotopic frequency below 230 Hz, ABF map acceptance was greater. In a SSD CI user, the reference side is the NH ear and the CI side is equivalent to the other (less deep) side. In the present study, most participants accepted the ABF mapping, irrespective of the angular insertion depth of E1.

For the binaural summation and squelch effects, significant improvements were observed with the ABF map relative to the CBF map. These improvements were also reasonably large in magnitude: the mean advantage for the ABF map over the CBF map was 0.65 dB SNR for summation and 0.94 dB SNR for squelch. It is not immediately clear why no significant effect of map type was observed for the SRM effect. The squelch and summation effects are both calculated by comparing aided versus unaided listening conditions. That is, they measure the performance gain with the addition of a CI. The addition of a CI with a map that provides a greater similarity of sound representation to the NH ear obviously leads to improved performance relative to the addition of a CI with a map providing less “normal” sound representation. In contrast, in the SRM test the participant is aided in both cases. SRM is calculated as the difference in performance when the masker and target are spatially separated target versus when the masker is co-incident with the target. The same map is used in both spatial configurations. As the benefits of SRM are largely derived from interaural timing differences (ITD) in the lower frequencies, it is possible that by raising the lower frequency limit as we have done here, ITDs are partially compromised.

In future studies, pitch matching experiments may be an alternative measure to evaluate perceptual matching between the CI and the NH ear in SSD users. This would be a key piece of evidence that the ABF technique provides frequency band allocations that better align with the natural tonotopic frequency distribution of the cochlea (i.e., a test tone is perceived as having the same pitch when delivered to the CI as when delivered to the NH ear). A limitation of the present study is the relatively small sample size (although it is the largest on this topic to date). Another potential limitation of this study is that the electrode frequency assignments used in ABF are based on the Greenwood function, which maps tonotopic frequency to cochlear position at the level of the OC. It has been argued that tonotopic frequency mapping may be better achieved at the level of the spiral ganglion (SG) (Stakhovskaya et al. [32]) although this is still a matter of debate in the CI field. The use of SG- based tonotopic frequencies would yield systematically different electrode frequency assignments in ABF. This study involved participants who received lateral wall electrode arrays, where the electrode contacts lie directly below the OC. With such arrays, it is likely that an OC-based map is appropriate. For other types of arrays such as those with a perimodiolar placement, an SG-based map may be more appropriate.

ABF is now routinely performed in our clinic. As such, more data is being continuously generated with both experienced and newly implanted CI recipients. Integrating ABF into the clinical routine is dependent upon close collaboration between the radiological imaging department and our unit.

## Conclusion

Compared to maps generated via conventional fitting techniques, maps generated via anatomy based fitting (ABF) improve speech perception in noise, binaural summation, and squelch effects in experienced CI users with SSD. Users’ self-perceived sound quality was similar – and usually high – with both ABF and CBF maps.
